# Fruit bats adjust their decision-making process according to environmental dynamics

**DOI:** 10.1186/s12915-023-01774-0

**Published:** 2023-11-29

**Authors:** Goni Naamani, Nitzan Shahar, Yoav Ger, Yossi Yovel

**Affiliations:** 1 School of Zoology, Faculty of Life Sciences, Tel Aviv University, Tel Aviv, 6997801 Israel; 2 Sagol School of Neuroscience, Tel Aviv University, Tel Aviv, 6997801 Israel; 3 The School of Psychological Sciences, Tel Aviv University, Tel Aviv, 6997801 Israel

**Keywords:** Decision making, Reinforcement learning, Bats, Volatility

## Abstract

One of the main functions of behavioral plasticity lies in the ability to contend with dynamic environments. Indeed, while numerous studies have shown that animals adapt their behavior to the environment, how they adapt their latent learning and decision strategies to changes in the environment is less understood. Here, we used a controlled experiment to examine the bats’ ability to adjust their decision strategy according to the environmental dynamics. Twenty-five Egyptian fruit bats were placed individually in either a stable or a volatile environment for four consecutive nights. In the stable environment, two feeders offered food, each with a different reward probability (0.2 vs. 0.8) that remained fixed over two nights and were then switched, while in the volatile environment, the positions of the more and the less rewarding feeders were changed every hour. We then fit two alternative commonly used models namely, reinforcement learning and win-stay-lose-shift strategies to the bats' behavior. We found that while the bats adapted their decision-making strategy to the environmental dynamics, they seemed to be limited in their responses based on natural priors. Namely, when the environment had changed slowly, at a rate that is natural for these bats, they seemed to rely on reinforcement learning and their performance was nearly optimal, but when the experimental environment changed much faster than in the natural environment, the bats stopped learning and switched to a random decision-making strategy. Together, these findings exemplify both the bats’ decision-making plasticity as well as its natural limitations.

**Supplementary Information:**

The online version contains supplementary material available at 10.1186/s12915-023-01774-0.

## Background

In a dynamic and ever-changing world, prediction of the future is both challenging and crucial. The ability to accurately estimate the outcomes of specific actions is of immense evolutionary advantage, while failures in prediction can result in reduced fitness. In order to determine the appropriate actions, animals must first gather and filter information about their environment develop a model of that environment, and then apply beneficial decision-making processes [1, 2]. In order to make predictions in a changing environment, it is important both to update one’s current knowledge of the environment and, probably, also to adapt one’s information acquisition strategy to keep up with the rate of changes in that environment [1, 3, 4].

Previous studies have found that animals are able to adapt their information acquisition and decision strategies according to the environment. For example, bumblebees were found to rely more on social cues when the foraging task was more difficult [3], and woodpeckers were found to maximize their food intake by changing their assessment and decision-making processes based on the difficulty of the foraging task [5].

In this study, we examined the ability of fruit bats to learn and respond to changing environmental dynamics. Egyptian fruit bats (*Rousettus aegyptiacus*) are nocturnal mammals that feed mainly on fruits but also on leaves and nectar [6, 7].

Fruit bats must routinely contend with a changing environment because they forage on ephemeral resources whose availability changes at different rates [8, 9]. On the one hand, they experience slow seasonal changes in which their diet undergoes change over many months, while, on the other hand, they also experience faster changes on the order of a single night when a specific fruit tree is depleted. The environmental dynamics that the bats experience might also undergo change when conspecific density changes (e.g., increases) and fruit trees are depleted more rapidly. It thus seems that it would be highly beneficial for fruit bats to be able not only to follow the changes in their environment but also to adjust their learning rate or even learning strategy according to the volatility of that environment.

Here, we tested Egyptian fruit bats’ foraging decisions in a controlled laboratory experiment. Twenty-five bats were placed individually in an environment in which they could choose between two feeders with different reward probabilities. The bats were divided into two groups that differed in their temporal dynamics. One group experienced the task in a more stable environment, in which each feeder offered its same reward probability for two consecutive nights, before switching between the two. The other bats experienced the same setup, but in a highly volatile environment, in which the reward probabilities changed every hour, probably faster than any resource change that these bats might encounter in the wild. The goal of this latter paradigm was to determine the upper limit of the bats’ ability to assess a changing environment.

We used two different learning and decision-making models to assess the bats’ decision-making process. (1) Reinforcement-learning models suggest that animal (including human) learning entails updating the values of previous actions, based on their outcomes [10–14]. Temporal-differences learning (e.g., Q-learning) is a form of reinforcement learning in which the learner has no prior knowledge on the probability of reward [10–12, 15–17]. Here, the learner follows and updates state-action values (known as *Q*-values), which are associated with the predicted values for each possible action given a certain state. *Q*-values are updated following each action based on a prediction-error (*δ*), which is defined as the difference between the action’s value prediction and the observed reward. The updating of the action’s *Q*-values is scaled based on a learning rate (*α*). Specifically, learning rates control the influence of past experience with low learning rates suggesting an integration of many previous trials, and high learning rates suggesting an influence of the most recent trials.

The optimal learning rate (*α*) depends on the rate of change in the environment, which is often referred to as its volatility. In a volatile environment, information must be updated more often than in a less volatile one in order to enable more accurate predictions. In a Q-learning framework, giving more weight to recent experience is equivalent to relying on a higher learning rate. In a stable environment, in contrast, distant previous experience can still be predictive and thus should be considered when making decisions, which is equivalent to using a lower learning rate. In general, integrating information derived over a longer period helps to avoid the effect of misleading (noisy) extreme values, whereas considering mostly recent outcome history is more optimal when the environment is volatile [10, 11, 14, 18].

Although the learning rate is key to dealing with a dynamic environment when using reinforcement learning, to the best of our knowledge, the effect of environmental volatility on learning has never been studied before in non-human animals. Little is known about animals’ ability to estimate the volatility of the environment and to adjust their learning rate accordingly or to switch their learning strategy in order to improve their updating of information. In this study, we therefore examined whether reinforcement learning could explain the bats’ behavior. Individual bats’ learning rates were estimated by fitting a reinforcement learning model to each bat based on its choices. We hypothesized that, if they use reinforcement learning, the bats exhibit different learning rates under different environmental volatilities.

(2) We also compared the bats’ behavior to a Win-Stay-Lose-Switch model (WSLS). According to this simple decision-making strategy, when a choice results in a favorable outcome (win), it increases the likelihood of sticking with the same choice (stay), and, conversely, if the outcome is unfavorable (lose), it prompts a shift to explore alternative options. Unlike reinforcement learning, the degree of surprise derived from the outcome does not play a role in the learning and decision-making processes.

Our findings suggest that bats rely on reinforcement learning in a stable environment and adjust their decision-making strategy based on the environment’s volatility. We also provide new insights into the limits of temporal learning.

## Results

Wild-caught bats were placed individually in a tent for four nights together with two feeders that secreted mango juice as a reward when they landed on them. One feeder was more rewarding than the other, with a reward probability of 0.8, while the other feeder had a reward probability of only 0.2. A total of 25 bats were allowed to individually explore one of the two different environmental dynamics for four nights (each). Fifteen bats experienced the stable environment, in which the rewarding and the less rewarding feeders remained in the same positions for two consecutive nights before switching their positions. Ten bats experienced the volatile environment, in which the positions of the rewarding and the less rewarding feeders were switched every hour (Fig. 2).

The bats performed an average of 120.3 ± 59.2 landing-trials per night, with no significant difference between the two groups (*P* = 0.47, unpaired two sample *T*-test, all results are presented as mean ± SD).

We used a reinforcement learning model to assess the bats’ learning rates in the two environments. We tested two models: a model without choice perseveration, with the learning rate (*α*) and an inverse temperature (*β*) as free parameters, and a model with choice perseveration that also included two additional parameters that represented the bats’ perseveration behavior (see the “Methods” section and [19, 20].

The data from all four nights of phase 2 (the learning phase) of the experiment were used to estimate the bats’ learning rate, using three different assumptions in each of the two above-noted models:

1. *Q*-values were initialized to zero between nights, thus assuming that the bats began learning from scratch each night.
2. *Q*-values decayed between nights, thus assuming that the bats would partially forget their previous night’s experience. To this end, a third reducing parameter was added to the model, simulating the “decay” factor between 0 and1. The decay factor was multiplied by the previous night’s *Q*-values, in which a higher decay factor decreased the *Q*-values, representing a more forgetful behavior. The decay factor was fitted to each bat individually using the same approach, together with α and β (see above)
3. The learning rate was estimated without adjusting the *Q*-values between nights, thus assuming that the bats had fully remembered their experience from the previous night.

In total, we tested six reinforcement learning models (three models without choice perseveration and three models with choice perseveration).

A comparison of the models’ BIC showed that the model assuming partial retention memory between consecutive nights, without perseveration, provided a better fit of the data for all bats, and we thus report the results for this model and used this model for the rest of the analysis (see Table 1, Additional file 1: Table S1, Table S2, which also provide the results for the AIC). In the rest of the study, we estimated the *α*, *β*, and the memory decay factor for each night separately, assuming partial retention memory between nights, without perseveration.

**Table 1:** Model parameters for the GLMM testing the effects of the environment on the learning rate and success rate. Environment and night as fixed effects, with a random effect of the individual bats, link function with identity

**Alpha**	**AIC**	**BIC**	**LogLikelihood**	**Deviance**				
	− 23.08	− 8.08	17.538	− 35.076				
	**Fixed effects coefficients (95% CIs):**							
	**Name**	**Estimate**	**SE**	**tStat**	**DF**	***P* value**	**Lower**	**Upper**
	**Intercept**	0.35	0.07	5.00	86	0.00	0.21	0.49
	**Volatile environment**	− 0.25	0.11	− 2.21	86	0.03	− 0.47	− 0.02
	**Night (1 to 4)**	− 0.05	0.02	− 2.67	86	0.01	− 0.09	− 0.01
	**Volatile environment: night**	0.07	0.03	2.10	86	0.04	0.00	0.13
**Success rate**	**AIC**	**BIC**	**LogLikelihood**	**Deviance**				
	128.93	143.93	− 58.46	116.93				
	**Fixed effects coefficients (95% CIs):**							
	**Name**	**Estimate**	**SE**	**tStat**	**DF**	***P* value**	**Lower**	**Upper**
	**Intercept**	− 0.38	0.19	− 2.00	86	0.05	− 0.75	0.00
	**Stable environment**	0.48	0.24	1.97	86	0.05	− 0.01	0.96
	**Night (1 to 4)**	0.05	0.06	0.91	86	0.36	− 0.06	0.17
	**Stable environment: night**	0.09	0.08	1.19	86	0.24	− 0.06	0.24

### Environmental effect on learning

**Fig. 1:**
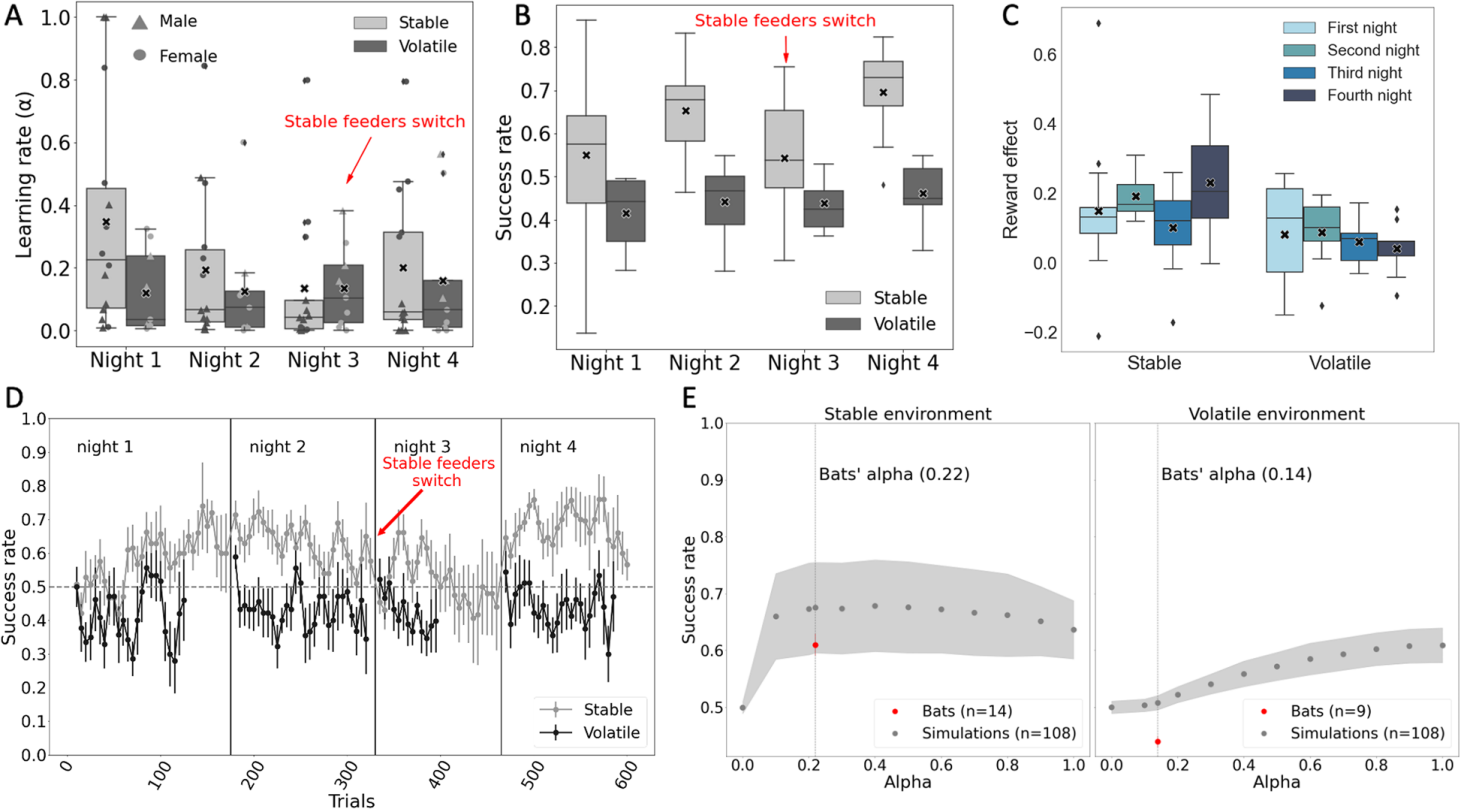
Bats adjust their foraging decision-making according to environmental volatility. In all panels, the boxes represent the area between the first quartile and the third quartile, with the median lines marked inside the boxes and the means marked with X. The lines from the boxes extend to the lowest/highest data points within 1.5 of the distance between the upper and lower quartiles. Circles and triangles represent females and males respectively. In panels A-C: stable: in nights 1-2: *n*= 14 bats, an in nights 3-4: *n*= 13 bats; volatile: *n*= 9 bats. **A** Both environment and night number had a significant effect on the learning rate (nights 1–2: *n* = 11 males, 12 females, nights 3–4: *n* = 11 males, 11 females). **B** Bats in the stable environment were more successful than bats that experienced the volatile environment. **C** The nightly reward effect on the stay probability for the volatile and the stable environments. **D** Bats that experienced the stable environment increased their success rate from the first to the second night, success rate decreased on the third night, and increased again in the fourth night. Success rate of bats that experienced the volatile environment did not change over the nights and was always close to chance (0.5). **E** Left—Stable environment simulations. A learning rate of 0.4 gave the optimal success rate. Right—Volatile environment simulations. A learning rate of 1 gave the optimal success rate. The estimated learning rate of the real bats is depicted by a dashed vertical line in both panels with the success rate of the bats shown as a red dot

Both environments (stable vs. volatile) and the night-number (1–4), as well as the interaction between them, had a significant effect on the learning rate (Fig. 1A, *P* = 0.03, *P* = 0.009 and *P* = 0.04, for the effects of environment, night, and their interaction respectively, mixed-effects generalized linear model—GLMM—with *α*—the learning rate set as the explanatory parameter, the environment and night as fixed effects, with a random effect of the individual bats, see Table 1). The average learning rate in the stable environment was significantly higher than in the volatile environment 0.22 ± 0.28 vs 0.14 ± 0.17, respectively. When excluding the third night (in which the stable feeder’s position was switched and *α* dropped accordingly), the difference was even more significant, and the two groups’ alphas were 0.25 ± 0.29 and 0.13 ± 0.18 for the stable and volatile environments, respectively. The average decay factor, a parameter that simulated the forgetful behavior of the bats between nights, was 0.33 ± 0.42 vs 0.55 ± 0.41 in the stable and the volatile environment, respectively.

### Foraging efficiency

The above analysis had revealed environment-dependent differences in learning rates. Next, we therefore sought to further understand to what extent these differences also translated to differences in overall gains. We therefore defined a bat’s success rate as the proportion of decisions that led to reward. Bats in the stable environment were significantly more successful in foraging than bats in the volatile environment (Fig. 1B, D, the success rate was 0.61 ± 0.08 vs. 0.44 ± 0.06 on average, respectively, *P* = 0.05, estimate = 0.48, GLMM with the success rate as the explanatory parameter, with a logit link function, and the other parameters as above, see Table 1). These results suggest that while the bats in the stable environment had learned the more rewarding feeder, the success rate of the bats that experienced the volatile environment did not significantly differ from chance (Fig. 1B, D, *P* = 0.15, binomial test), and did not improve over consecutive nights (*P* = 0.26, one-way repeated measures ANOVA, with the success rate of the volatile bats as the explanatory parameter and the night-number (1–4) as the explanatory factor).

We next used a simulation to estimate the optimal learning rate for each environment (i.e., the learning rate that provides the highest foraging success; the “Methods” section, Fig. 1E). Our results reveal that, in the stable environment, the bats’ performance was not far from the optimal enabled by reinforcement learning, while in the volatile environment, they fell far below the optimal. In the stable environment, the maximal success rate in the simulation (obtained for alpha = 0.4) was 0.68 ± 0.08 in comparison to 0.61 ± 0.08 in the real bats. This difference was not significant on any of the nights (first night: *P* = 0.14, 0.55 ± 0.18, second night: *P* = 0.32, 0.65 ± 0.11, third night: *P* = 0.12, 0.54 ± 0.12, fourth night: *P* = 0.55, 0.7 ± 0.1 for the real bats, vs. 0.68 ± 0.08 for the simulated bats, we compared the real bats’ success rate to 108 simulations, see the “Methods” section). The fact that the bats’ success was not different from that expected when using reinforcement learning with an equivalent learning rate strengthens our conclusion that they were employing some type of reinforcement learning.

In the volatile environment, the maximal simulated success rate (obtained for alpha = 1) was 0.61 ± 0.03 in comparison to a significantly lower rate in the real bats 0.44 ± 0.06 (first night: *P* = 0.009, 0.42 ± 0.08, second night: *P* = 0.009, 0.44 ± 0.09, third night: *P* = 0.009, 0.44 ± 0.06, fourth night: *P* = 0.009, 0.46 ± 0.07, for the real bats, vs. 0.61 ± 0.03 for the simulated bats, for 108 simulations). This significant difference between the bats’ and the model’s performance (even when fitting the behavior to the model) suggests that the bats were not using reinforcement learning in the volatile environment, which led us to examine another model (below).

### Reward effect on stay probability

A well-established approach that enables the uncovering of learning over consecutive nights is to examine the effect of the outcome (i.e., reward vs. no reward) on the bats’ staying behavior, i.e., their probability of choosing the same feeder in two consecutive trials (the “Methods” section). The overall effect of reward on stay probability was significantly higher than zero in both groups (*P* = 3.13E − 12, *P* = 0.0001 for the stable and volatile environment, respectively, one sample, one-sided *T*-test, see the “Methods” section). However, the reward effect showed opposite patterns in the two different environments: steadily (and significantly) decreasing in the volatile environment, while remaining the same in the stable environment (Fig. 1C, *R* =  − 0.98, *P* = 0.02 and *R* = 0.6, *P* = 0.4 for the volatile and stable environments, respectively, Pearson’s correlation test for the correlation between the reward effect and night number). These results suggest that the bats’ behavior in both environments was sensitive to reward delivery. However, while the bats in the stable environment had clearly learned which feeder was more rewarding, the bats in the volatile environment appear to have learned that there was no environmental regularity and they probably switched to using a different strategy, which we further discuss below.

### Win-Stay-Lose-Shift (WSLS) model

We also compared the likelihood of the reinforcement learning model to a simple Win-Stay-Loose-Shift (WSLS) strategy (the “Methods” section). WSLS is a straightforward decision-making model. It operates on the principle that when a choice results in a favorable outcome (win), it increases the likelihood of sticking with the same choice (stay). Conversely, if the outcome is unfavorable (lose), it prompts a shift to explore alternative options. The WSLS and the reinforcement learning models had similar likelihoods for both the volatile and stable environments (74.29 ± 37.67 vs. 76.67 ± 33.88 and 67.14 ± 40.31 vs. 64.24 ± 39.13, respectively. There was no significant difference between the likelihood of the models in either of the environments: *P* = 0.46 and *P* = 0.2, paired sample *T*-test).

In contrast to the similar likelihoods, the success rates predicted by the WSLS (after fitting their parameters to the data) significantly differed from those observed in reality. Success rate in the simulations was significantly higher than in the real bats in the volatile environment (0.52 vs 0.44 ± 0.07, respectively, *P* = 0.01, we compared the bats’ success rate to 100 simulations, see the “Methods” section) and significantly lower than reality in the stable environment (0.52 vs 0.61 ± 0.09, respectively, *P* = 0.01, for 100 simulations).

## Discussion

In this study, we examined how animals, specifically fruit bats, adjust their information acquisition and decision-making strategy in environments with different temporal dynamics. We found that while fruit bats can clearly learn the dynamics of their environment, their learning seems to be restricted to biologically relevant temporal dynamics. Specifically, we found that when the environment changed very fast (every hour) the fruit bats did not elevate their learning rate to keep up with the rate of change. In the wild, Egyptian fruit bats feed on fruit and nectar-providing trees, which offer food for time periods of several days–weeks. A switch in resource availability occurring after 2 days, such as that which the stable group experienced in our study, is thus within the natural range experienced by this species in the field, and, accordingly, the bats learned it, and their performance was comparable to an optimal reinforcement learning agent. The bats’ success was thus not significantly different from the prediction of the best reinforcement-learning model.

Some indirect evidence suggests that fruit bats in the wild are also able to adjust their foraging-decision strategy. In a previous study, it was shown that in urban environments, which are characterized by faster temporal dynamics, bats switch between foraging trees much more often than in rural environments, doing so on an almost nightly basis [8]. The advantage of this behavior is not clear, but one explanation could be the need to increase their environmental update rate. Although recent study has shown that urban bat pups are faster learners than rural pups [9], that study only compared bat populations and did not relate to the possibility that individuals are able to adjust their learning rate, as we examined here.

In comparison to the stable environment, the volatile environment, which changed every hour, offered a temporal pattern that is probably never encountered in the wild by these bats. We note that although the bats might occasionally experience nectar trees that become depleted within a few hours, they probably never experience a switching environment in which an entire tree is depleted and then replenished within 2 h. Counter to our expectation, rather than increasing their learning rate in the more volatile environment, the bats significantly decreased it, implying that they simply could not counter this swift rate of change (see more below).

It has been shown in humans that they will adjust their learning rate according to environmental volatility. While we find that such adjustments are probably limited by priors about environmental dynamics. We cannot determine whether these priors are innate or learned. Some animals have been shown to sample the environment in order to update their priors. Woodpeckers, for example, have been shown to change their decision-making process in a foraging task based on task difficulty: in the more difficult task, when uncertainty was higher, they spent more time gathering sufficient information before making a decision [5].

Learning rates also seem to be species-specific within bats. Goldshtein et al. [21] showed that nectarivorous bats that forage in an environment with much faster dynamics, where nectar is routinely depleted and replenished within hours, exhibit much faster learning.

Our current findings suggest that a reinforcement-learning model could explain the behavior of the bats in the stable environment, but what strategy did the bats in the volatile environment employ? Both models that we tested (reinforcement-learning and WSLS) had a similar likelihood, but both predicted significantly higher performance (success) than that demonstrated by the actual bats. We thus suggest that the bats in the volatile environment adopted a random selection strategy that located their performance at chance level. This is somewhat surprising, because even a simple pure Win-Stay-Lose-Shift strategy could have yielded a better performance (Additional file 1: Figure S1). One reason not to use a pure WSLS strategy (in which the animal either stays 100% of the time or shifts 100% of the time, based on reward) was suggested by Lyu et al. [22] who noted that shifting resources (in our case the feeders) also comes with a cost and thus might prevent animals from using a WSLS strategy if the environment is highly unstable.

Note that the bats in the volatile environment seemed initially to try learning, as can be understood from the effect of reward on their stay probability. Any learning model should increase the probability of an action that leads to reward. Consequently, a reward should have a positive effect on the probability of repeating the action that led to this reward, which in our task meant choosing the same feeder again in case it proved to be profitable. The overall effect of reward on the stay probability was significantly higher than zero in both groups, meaning that the bats were more likely to return to the same feeder after receiving a reward. However, while the effect of reward remained roughly the same (with a slight tendency to increase) in the stable environment, it constantly decreased in the volatile environment, reaching nearly zero on the last night, suggesting that the bats were gradually giving up on learning.

## Conclusions

Our findings suggest that bats possess behavioral plasticity, allowing them to adjust their information acquisition and decision-making strategy according to the environment’s dynamics. They also indicate that such plasticity might however be bounded and restricted to ecologically plausible environments only.

## Methods

### Study species and housing

The study comprised 25 Egyptian fruit bats (*Rousettus aegyptiacus*) (12 females: 8 adults, 4 juveniles; and 13 males: 11 adults, 2 juveniles, see Table 2). The adult bats were captured in a natural colony in central Israel (Herzliya) in December 2019 and were housed together with an already existing colony of Egyptian fruit bats. The juvenile bats were either born in our captive colony or brought to the colony with their mothers at a very early age.

**Table 2:** The 25 bats that participated in the study. Asterisks mark bats that were born in the captive colony. The table indicates which environmental condition was experienced by each bat

Subject number	Name	Origin	Sex	Age	Environment
1	Shraga	Herzliya	M	Adult	Stable
2	Yossi	Herzliya	M	Adult	Stable
3	Gimel	Herzliya	M	Adult	Volatile
4	Lamed	Herzliya	M	Adult	Stable
5	Aein	Herzliya	M	Adult	Volatile
6	Eight	Herzliya	M	Adult	Stable
7	Tzadi	Herzliya	M	Adult	Stable
8	Zurik	Herzliya	M	Adult	Volatile
9	Amos	Herzliya	M	Adult	Stable
10	Four	Herzliya	M	Adult	Stable
11	Arrow	Herzliya	F	Adult	Stable
12	Slash	Herzliya	F	Adult	Volatile
13	W	Herzliya	M	Adult	Stable
14	Percent	Herzliya	F	Adult	Volatile
15	LT	Herzliya	F	Adult	Stable
16	w-pup	Herzliya	F	Juvenile	Stable
17	2 dots	Herzliya	F	Juvenile	Stable
18	4-pup	Countryside	F	Juvenile	Stable
19	u-pup	Herzliya	F	Juvenile*	Stable
20	mirrored V	Herzliya	M	Juvenile	Volatile
21	mirrored R	Herzliya	M	Juvenile*	Stable
22	Lynn Hill	Herzliya	F	Adult	Volatile
23	Rosalind	Herzliya	F	Adult	Volatile
24	Ada	Herzliya	F	Adult	Volatile
25	Simone	Herzliya	F	Adult	Volatile

The colony (4 × 2 × 2.9 m^3^) comprised a total of 30 bats. They were kept under a 12:12 light to dark cycle, at a stable temperature of 24 ± 2 °C.

### Experimental setup

**Fig. 2:**
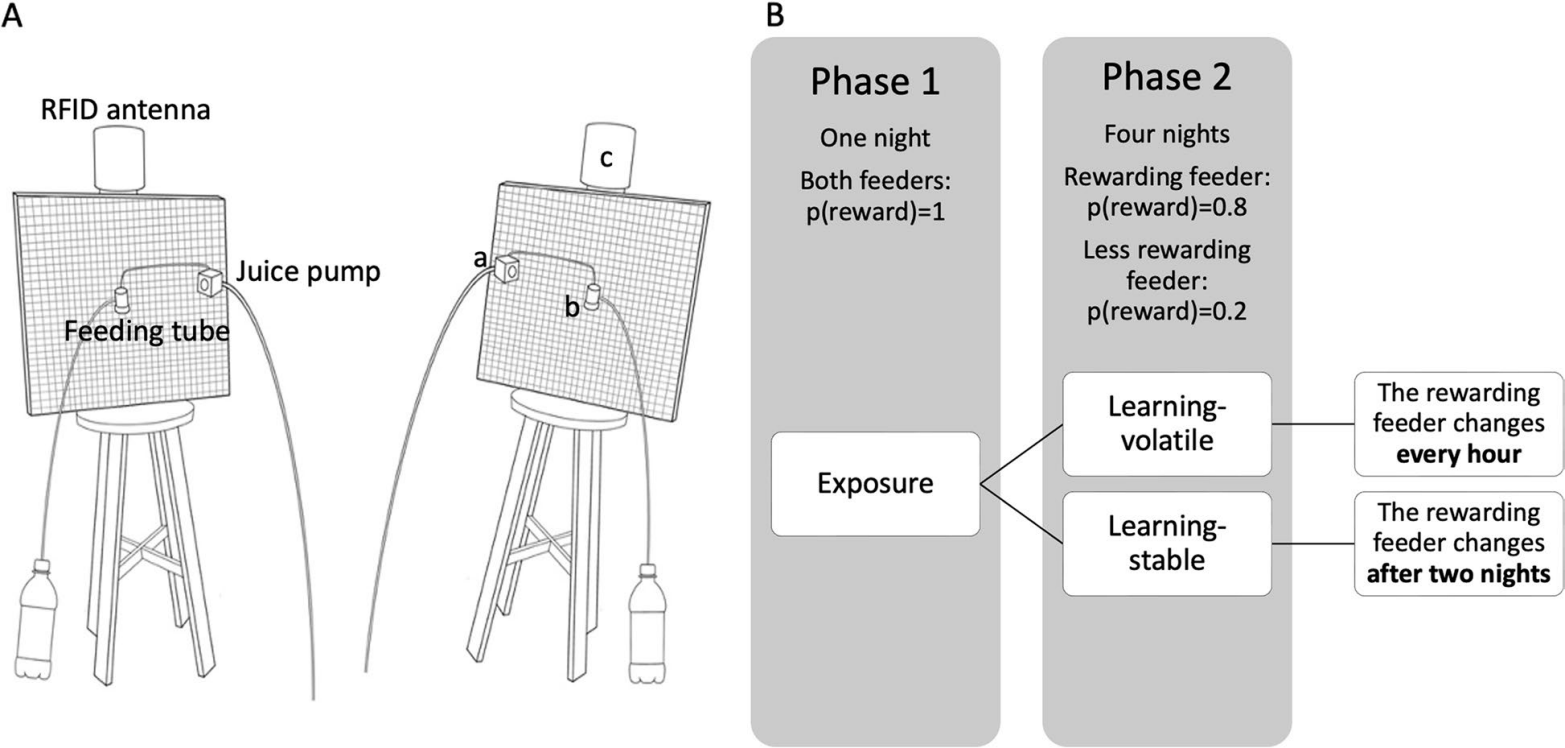
**A** Experimental setup. (a) Two feeders with landing platforms and RFID antennas. (b) A—juice pump, B—feeding tube, C—RFID antenna. The bottle on the side collected juice spillover, allowing us to quantify drinking. **B** The experimental procedure—phase 1: both of the feeders reward with *p*(reward) = 1. Phase 2: the bats were divided between a stable and a volatile environment. The rewarding feeder (*p*(reward) = 0.8) and the less rewarding feeder (*p*(reward) = 0.2) changed either every hour (volatile environment) or after 2 nights (stable environment)

The experiments took place in two identical tents (2.4 × 2.4 × 1.85 m^3^) in a controlled temperature room (24 ± 2 °C), next to the captive bats’ colony, allowing the bats in the tents to hear the sounds from the colony and thereby reduce stress. The floor underneath each tent was covered with 25 pieces of 50 × 50 × 1 cm soft foam mat, to prevent bat injury in case of a fall. The tent walls were covered with black felt from about 1 m above the floor to the tent top, allowing the bats to hang anywhere. A 30 × 30 cm^2^ plastic net was placed at the top of the tent to enable perching. Each tent held two feeders, placed one meter apart. Each feeder (see Fig. 2A) contained a 50 × 50 × 1 cm^3^ vertical wooden platform covered with felt and a plastic net. A pump was installed on the side of the wooden square, with a 5-mm pipe that ended in a 10-ml tube. Another pipe was connected to the tube, allowing a max of 6 ml of juice to accumulate in the tube. The pumps were programmed to secret 3 ml of mango juice whenever a bat landed on the platform. Detection of landing was based on RFID. All landings were logged to a computer. A GeoVision camera located on the floor filmed the two feeders during the experiments.

### Experimental procedure

The experiments consisted of two phases: the exposure phase and the learning phase (see Fig. 2B). All experiments took place at night, the bats’ natural activity time, from around 16:00 to around 9:00 the next day, after the same number of hours since being fed. The bats were kept in the captive colony in between experiments. In addition to the mango-juice feeders, a bowl of water was permanently available in the tents.

#### Phase 1: Exposure

The goal of this phase was to familiarize the bats with the experimental setting and to ensure that they had learned to feed from the feeders. To reduce the bats’ stress, the exposure phase was done using pairs of bats. The bats were kept in the experimental tent for one night and both feeders provided a reward of 3 ml of mango juice for each landing.

#### Phase 2: Learning

In this phase, we measured the bats’ learning rate. Each bat was kept alone in the same experiment tent for four consecutive nights. One of the feeders was more rewarding (hereafter the “rewarding feeder”) with a probability of 0.8 of providing a reward with each landing, while the other feeder was less rewarding (hereafter the “less rewarding feeder”), with a probability of only 0.2 of providing a reward. The first position of the rewarding feeder was on the right side of the tent for half of the bats and on the left for the other half. The bats were randomly allocated to either a stable or a volatile environment. In the stable environment, the rewarding and the less rewarding feeders remained in the same positions for two consecutive nights, after which their positions were switched. In the volatile environment, the position switch between the rewarding and the less rewarding feeders was systematically applied every hour (i.e., R-L-R-L).

Fifteen bats (10 adults and 5 juveniles) were exposed to a stable environment, and 10 bats were exposed to a volatile environment. During the daytime, the bats were returned to their colony.

### Analysis

A trial was defined as a bat landing on a feeder—either when switching from one feeder to the other or when landing on the same feeder, when at least three seconds had passed from the time of previously leaving that feeder. We extracted the following information for each bat: the time of each landing, the chosen feeder in each trial, the reward the bat received (0 for no reward or 1 if it received a reward) in each trial, and the total number of trials.

#### Estimating the bats’ learning rates- Reinforcement-learning models

We tested two models—a model without choice perseveration and a model with choice perseveration, as explained below, using MATLAB scripts.

We used maximum-likelihood estimation using the Matlab function “optimoptions” to estimate bats’ individual parameters. For each bat, we used 50 starting points that were randomly chosen by the computer. We used the best fitting parameters as an estimate for the bats' latent learning parameters. We compared the models’ fit using a BIC score.

#### Model without perseveration

We used a Q-learning model to calculate the learning rate (*α*). We simulated *Q*-values for each action on each step and the probability of choosing each action according to the SoftMax policy (see Eq. 1) [15, 23], where *β* is the inverse temperature parameter, i.e., the parameter that determines the sensitivity of the choice probabilities to the difference in values. Large values of *β* make the choice more sensitive to the values difference, while low values of *β* make the choice less sensitive to the difference in values [15, 23].

1 $$P\left(a\left(t\right)=1\right)=\frac{exp(\beta {*Q}_{a,t}) }{\mathit{exp}(\beta {*Q}_{a,t}) +exp(\beta {*Q}_{b,t})}$$

where ${Q}_{i}$ is the *Q*-value for the chosen action i (*a* or *b*), *β* is the inverse temperature parameter, and *t* is the index of the trial.

*Q*-values are updated based on the outcome (see Eq. 2).

2 $${Q}_{i}(t+1)={Q}_{i}(t)+\alpha *(Reward(t)-{Q}_{i}(t))$$

where ${Q}_{i}$ is the *Q*-value for the chosen action *i*, and *t* is the index of the trial. *α* is the learning rate. The *Q*-value of the unchosen action is not updated [15].

#### Model with choice perseveration

The model with perseveration includes a learning rate (*α*) and inverse temperature (*β*) as in the model without perseveration, together with two additional parameters that represent the bats’ perseveration behavior (the perseveration rate and perseveration exponent). This model keeps track of choice perseveration values (${p}_{val}$) for each action, which determines the perseveration strengths of each action (similar in spirit to the *Q*-values). The ${p}_{val}$ are initiated at 0.5 and updated after each trial according to the perseveration rate (${p}_{rate}$) free parameter (between 0 and 1), which determines the magnitude of updating. The updating increases the ${p}_{val}$ of the selected action toward 1 and the unselected action ${p}_{val}$ toward 0, regardless of the outcome (see Eq. 3). We also included an additional perseveration exponent (${p}_{exp}$) free parameter which determines how perseverative the bat is; note that the setting ${p}_{exp }=0$ is exactly like the RL model without perseveration. *Q*-values are updated similarly to the model without perseveration (see Eq. 2) [19, 20].

3 $$\begin{array}{c}p\left(a\left(t\right)=1\right)=\frac{\mathrm{exp}(\upbeta *{Q}_{a}+{p}_{exp}*{p}_{val\left(a\right)})}{\mathrm{exp}\left(\upbeta *{Q}_{a}+{p}_{exp}*{p}_{val\left(a\right)}\right)+\mathrm{exp}\left(\beta *{Q}_{b}+{p}_{exp}*{p}_{val\left(b\right)}\right)}\\ { \left(1\right) p}_{val\left(a\right)}=\left(1-{p}_{\mathrm{rate}}\right)* {p}_{val\left(a\right)}\\ \begin{array}{c}{p}_{val\left(b\right)}=\left(1-{p}_{\mathrm{rate}}\right)*{p}_{val\left(b\right)}\\ {\left(2\right) p}_{val\left(\mathrm{choice}\right)}={p}_{val\left(\mathrm{choice}\right)}+{p}_{rate}\end{array}\end{array}$$

where *a* is the action, *β* is the inverse temperature parameter, ${Q}_{i}$ is the *Q* value for the chosen action *i* (*a* or* b*), *P* exp is the perseveration exponent, *P* val is perseveration value, and *t* is the index of the trial).

#### Reward effect on stay probability

*Staying* was defined as returning to the feeder that had been visited in the previous trial. We estimated the *stay probabilities* (for each individual) in both the cases in which the bat had received a reward in the previous trial and in which it had not. To examine the effect of reward on the stay probability (which we term the “reward effect”), we subtracted the individual stay probability for no reward from that following reward (see Eq. 4).

4 $$\mathrm{Reward}\;\mathrm{effect}=P\left(\mathrm{staying}\vert\mathrm{reward}\right)-P\left(\mathrm{staying}\vert\mathrm{no}\;\mathrm{reward}\right)$$

#### Foraging efficiency

To determine bats’ foraging efficiency, we calculated their success rate. Success rate was defined as the proportion of actions that led to reward (see Eq. 5).

5 $$\mathrm{Success}\;\mathrm{rate}=\frac{\mathrm{number}\;\mathrm{of}\;\mathrm{rewarded}\;\mathrm{actions}}{\mathrm{all}\;\mathrm{actions}}$$

#### Reinforcement-learning simulations

To examine whether the bats employed an optimal learning rate that would maximize their success rate, we simulated data using a grid search separately for each environment and noted the resulting success rates. Specifically, we changed the learning rate gradually from 0 to 1 in steps of 0.1. For each learning rate value in the grid search, each agent was simulated using a *β* parameter that was sampled from the empirical *β* distribution estimated for the bats (i.e., the bats’ estimated *β* were used), separately for each environment. Since in the stable environment there were 13 bats each tested for four nights, and one bat that was tested for only two nights, this totaled on 54 bats’ estimated *β*. Each estimated *β* was used twice, thus simulating 108 agents in total in the stable environment for each learning rate. In the volatile environment, nine bats had been tested each for four nights, resulting in 36 estimated *β*. Each estimated *β* was used by three agents, thus simulating 108 agents in total in the volatile environment for each learning rate. The number of trials was 2000 in each simulation.

In our empirical setting, the stable group had a fixed reward probability for each feeder for two nights and a reversed probability for two additional nights; while the volatile group received reversal in the reward probability every hour. Bats in the stable environment performed on average 251 trials on nights 1–2 before the reversal in probability and 214 trials on nights 3–4 (after the reversal in probability). Thus, for the stable environment simulation, we used the same fixed reward probability as in our empirical settings for 251 trials, then switched the reward probability between the choices for 214 trials and returned this process until 2000 trials had been carried out. For the volatile environment, we switched the reward probability every 13 trials until reaching 2000 trials. This was done because in the volatile environment the bats had performed 13 trials on average within an hour, before the reward probabilities were switched. The simulations are equivalent to the no perseveration model without an adjustment of the *Q*-values between ‘nights’; i.e., we did not simulate forgetting.

#### Win-Stay-Lose-Shift (WSLS) model

Given the binary decision of the task, we modeled a WSLS rule-based strategy [24, 25]. Allowing for some deviation from the deterministic ruling, we used a two free parameter model—the Win-Stay probability ($${p}_{stay})$$, which is the probability that the bat returns to the same feeder in the next trial after receiving a reward from it in the previous trial, and the Lose-Shift probability ($${p}_{shift})$$, which is the probability that the bat shifts to the other feeder in the next trial after not receiving a reward from it in the previous trial (see Eq. 6).

6 $$p\left(a(t)=i\right)= \left\{\begin{array}{c}{p}_{stay} \\ {p}_{shift} \end{array}\right.\begin{array}{c}\mathrm{if }\\ \mathrm{if }\end{array}\begin{array}{c}{a}_{t-1}={a}_{i}\mathrm\;{ and }\;{r}_{t-1}=1 \\ {a}_{t-1}={a}_{i}\mathrm\;{ and }\;{r}_{t-1}=0\end{array}$$

The probability of choosing action *i* if action *i* had been chosen in the previous trial is either ${p}_{stay}$ or ${p}_{shift}$, depending on the previous reward. *a* is the action, *t* is the index of the trial, and *r* is the reward (1 or 0).

Parameter estimation for each bat’s decisions was done using maximum-likelihood with a self-written Python script.

#### WSLS simulations

For each environment (e.g., stable or volatile) and for each combination of WSLS model parameters (from 0 to 1 in steps of 0.1), we ran 100 simulations of 2000 trials each. The reward probability of each action was identical to those used in the real bat experiments. We averaged across the 100 simulations of each environment and parameter combination and calculated the success rate (Eq. 5).

#### Discarded data

Due to a mechanical problem in the feeding system, one of the bats (bat 18, the stable environment group) did not obtain any rewards during its third night in the experiment. Data from the third and fourth nights of this bat were therefore discarded. When examining the effect of reward on the stay probability, two bats (bat 1, stable environment group; and bat 8, volatile environment group) demonstrated a negative effect in three out of the four experimental nights. This suggests that they had failed to learn, and they were consequently also removed from the analysis.

#### Statistics

We used a mixed-effects generalized linear model—GLMM—with α—the learning rate set as the explanatory parameter, the environment and night as fixed effects, and subject ID as a random effect. To examine the effect of the environment (stable vs. volatile) on the learning rate, the model without the sex interaction produced a better fit (BIC =  − 8.08 for the model without sex interaction vs BIC =  − 7.05 for the model with sex interaction). To determine the effect of the environment on success rate, we used a GLMM, with the success rate set as the explanatory parameter, with a logit link function, and the other parameters as above. To determine the significance of the reward effect on stay probability, we used a one-sample one-sided *T*-test on each group (stable or volatile). To determine the nightly reward effect on stay probability, we used Pearson’s correlation of median reward effect with night number (1–4).

To determine the difference in success rate between the real bats and the simulations, we compared the real bats’ success rate to 108 reinforcement-learning simulations (with optimal alpha) and to 100 WSLS simulations with the same parameters as estimated for the bats and examined whether the bats’ success rate in each group was in the lowest or highest 5% of the simulations’ success rate within the same environment.

To compare between all the reinforcement models, we used BIC. BIC is considered to be more conservative than AIC and therefore more suitable for a small sample size in estimating model fit.

To compare between the reinforcement-learning model without choice perseveration and the Win-Stay-Lose-Shift model, we compared their log-likelihoods, since both models have the same number of free parameters. All the results are presented as mean ± SD.

### Supplementary Information

**Additional file 1: Table S1.** Models’ BIC comparison. **Table S2.** Models’ AIC comparison. **Figure S1.** WSLS model simulations’ success rate. 

## Data Availability

All data generated or analyzed during this study are included in this published article, its supplementary information files, and publicly available repositories. The datasets and code generated or analyzed during the current study are available in the Mendeley Data at https://doi.org/10.17632/4r6ydm6vgs.3. All the code used during this study is also available at https://github.com/goninaa/bats-adjust-their-decision-process.
