# Supplementary material for: Fruit bats adjust their decision-making process according to environmental dynamics
Source: BMC Biol. 2023 Nov 29;21:278. doi: 10.1186/s12915-023-01774-0 (PMC10687778; doi:10.1186/s12915-023-01774-0)
Supplement: Supplementary file 1 — Additional file 1: Table S1. Models’ BIC comparison. Table S2. Models’ AIC comparison. Figure S1. WSLS model simulations’ success rate. [file 12915_2023_1774_MOESM1_ESM.docx]

# Additional file 1

|  | | | | **Models without perseveration –**  **BIC** | | | **Models with perseveration –**  **BIC** | | |
| --- | --- | --- | --- | --- | --- | --- | --- | --- | --- |
| **Subject** | **Environment** | **Sex** | **Age** | **No memory (Q zeroed)** | **Partial memory (Q decayed)** | **Full memory (continuous Q)** | **No memory (Q zeroed)** | **Partial memory (Q decayed)** | **Full memory (continuous Q)** |
| 2 | Stable | M | Adult | 21.560 | 21.560 | 23.968 | 33.789 | 36.280 | 36.280 |
| 3 | Volatile | M | Adult | 21.825 | 21.793 | 23.696 | 34.032 | 35.905 | 35.908 |
| 4 | Stable | M | Adult | 19.661 | 19.661 | 20.965 | 30.675 | 31.936 | 31.941 |
| 5 | Volatile | M | Adult | 22.315 | 22.313 | 24.448 | 34.923 | 37.054 | 37.054 |
| 6 | Stable | M | Adult | 22.849 | 22.849 | 25.317 | 36.005 | 38.234 | 38.234 |
| 7 | Stable | M | Adult | 21.591 | 21.591 | 24.738 | 34.623 | 37.612 | 37.623 |
| 9 | Stable | M | Adult | 15.119 | 14.296 | 19.439 | 25.046 | 30.092 | 30.093 |
| 10 | Stable | M | Adult | 21.868 | 21.868 | 24.316 | 34.568 | 36.907 | 36.907 |
| 11 | Stable | F | Adult | 23.341 | 23.341 | 25.547 | 36.538 | 38.656 | 38.656 |
| 12 | Volatile | F | Adult | 22.298 | 22.262 | 24.967 | 35.023 | 37.708 | 37.711 |
| 13 | Stable | M | Adult | 22.079 | 22.066 | 23.686 | 34.492 | 36.048 | 36.048 |
| 14 | Volatile | F | Adult | 21.783 | 21.755 | 23.579 | 33.539 | 34.495 | 34.501 |
| 15 | Stable | F | Adult | 16.081 | 16.003 | 18.540 | 26.378 | 28.794 | 28.799 |
| 16 | Stable | F | Juvenile | 22.357 | 22.357 | 24.240 | 34.980 | 36.687 | 36.687 |
| 17 | Stable | F | Juvenile | 21.907 | 21.907 | 23.866 | 34.513 | 36.392 | 36.393 |
| 18 | Stable | F | Juvenile | 19.474 | 19.471 | 20.372 | 30.206 | 31.087 | 31.087 |
| 19 | Stable | F | Juvenile | 20.852 | 20.852 | 23.197 | 32.860 | 34.797 | 34.797 |
| 20 | Volatile | M | Juvenile | 22.503 | 22.503 | 24.442 | 35.447 | 37.236 | 37.242 |
| 21 | Stable | M | Juvenile | 17.597 | 17.591 | 19.364 | 26.214 | 29.006 | 29.006 |
| 22 | Volatile | F | Adult | 20.335 | 20.322 | 22.684 | 32.060 | 34.393 | 34.393 |
| 23 | Volatile | F | Adult | 20.623 | 20.618 | 22.514 | 32.280 | 33.959 | 33.966 |
| 24 | Volatile | F | Adult | 23.183 | 23.183 | 24.647 | 36.444 | 37.818 | 37.818 |
| 25 | Volatile | F | Adult | 19.599 | 19.599 | 21.886 | 30.847 | 33.187 | 33.187 |
| **Mean** |  |  |  | 20.904 | 20.859 | 23.062 | 32.847 | 34.969 | 34.971 |
| **SD** |  |  |  | 2.161 | 2.271 | 2.025 | 3.265 | 2.980 | 2.980 |
| **SE** |  |  |  | 0.451 | 0.473 | 0.422 | 0.681 | 0.621 | 0.621 |

**Table S1:** Models’ BIC comparison showed that the partial memory (Q-decayed) model without perseveration provided a better fit of the data.

|  | | | | **Models without perseveration –**  **AIC** | | | **Models with perseveration –**  **AIC** | | |
| --- | --- | --- | --- | --- | --- | --- | --- | --- | --- |
| **Subject** | **Environment** | **Sex** | **Age** | **No memory (Q zeroed)** | **Partial memory (Q decayed)** | **Full memory (continuous Q)** | **No memory (Q zeroed)** | **Partial memory (Q decayed)** | **Full memory (continuous Q)** |
| 2 | Stable | M | Adult | 199.356 | 199.356 | 655.291 | 187.680 | 632.016 | 632.016 |
| 3 | Volatile | M | Adult | 239.915 | 236.174 | 605.189 | 235.085 | 587.304 | 588.107 |
| 4 | Stable | M | Adult | 153.376 | 153.376 | 290.619 | 155.920 | 285.715 | 286.504 |
| 5 | Volatile | M | Adult | 260.256 | 259.975 | 748.534 | 264.220 | 751.511 | 751.511 |
| 6 | Stable | M | Adult | 243.305 | 243.305 | 825.965 | 232.990 | 693.704 | 693.704 |
| 7 | Stable | M | Adult | 148.416 | 148.414 | 700.267 | 152.443 | 651.548 | 654.871 |
| 9 | Stable | M | Adult | 21.107 | 15.359 | 151.963 | 19.032 | 143.429 | 143.469 |
| 10 | Stable | M | Adult | 199.615 | 199.615 | 669.368 | 203.538 | 637.607 | 637.607 |
| 11 | Stable | F | Adult | 322.992 | 322.992 | 965.128 | 327.031 | 927.700 | 927.700 |
| 12 | Volatile | F | Adult | 225.356 | 221.416 | 844.773 | 212.385 | 790.281 | 791.235 |
| 13 | Stable | M | Adult | 253.674 | 252.060 | 561.697 | 256.188 | 548.329 | 548.329 |
| 14 | Volatile | F | Adult | 232.560 | 229.484 | 565.081 | 181.733 | 288.242 | 288.986 |
| 15 | Stable | F | Adult | 39.147 | 37.805 | 124.035 | 42.347 | 122.446 | 122.682 |
| 16 | Stable | F | Juvenile | 263.766 | 263.766 | 670.041 | 267.757 | 617.812 | 617.822 |
| 17 | Stable | F | Juvenile | 212.989 | 212.989 | 560.567 | 216.765 | 542.061 | 542.277 |
| 18 | Stable | F | Juvenile | 162.299 | 162.085 | 251.974 | 166.427 | 254.037 | 254.037 |
| 19 | Stable | F | Juvenile | 167.345 | 167.343 | 531.426 | 168.242 | 430.063 | 430.063 |
| 20 | Volatile | M | Juvenile | 239.849 | 239.849 | 625.665 | 241.624 | 579.314 | 581.068 |
| 21 | Stable | M | Juvenile | 81.991 | 81.779 | 192.603 | 42.308 | 145.857 | 145.857 |
| 22 | Volatile | F | Adult | 149.946 | 148.956 | 476.340 | 151.795 | 469.360 | 469.360 |
| 23 | Volatile | F | Adult | 180.962 | 180.519 | 459.342 | 184.998 | 417.598 | 418.938 |
| 24 | Volatile | F | Adult | 286.115 | 286.022 | 590.508 | 286.705 | 562.153 | 562.153 |
| 25 | Volatile | F | Adult | 126.266 | 126.266 | 387.541 | 122.875 | 377.766 | 377.766 |
| **Mean** |  |  |  | 191.765 | 190.822 | 541.475 | 187.830 | 498.081 | 498.524 |
| **SD** |  |  |  | 75.655 | 76.089 | 224.664 | 78.164 | 217.099 | 217.201 |
| **SE** |  |  |  | 15.775 | 15.866 | 46.846 | 16.298 | 45.268 | 45.289 |

**Table S2:** Models’ AIC comparison showed that the no memory (Q-zeroed) model with perseveration provided a better fit of the data.

According to the AIC, the no memory model with choice perseveration had the best fit of the data. The learning rate predicted by this model in the stable environment was similar to that of the best model according to the BIC (α=0.25±0.33) while the learning rate estimated in the volatile environment had a higher learning rate (α=0.44±0.37). This was in-line with the hypothesis that bats will adapt learning to environmental volatility, but as we mention in the main text, the bats’ performance was significantly lower than that predicted based on simulations (see Figure 1E) and thus we find it unlikely that the bats used this model. The next best fit model according to the AIC was the partial memory without perseveration model (the same as the BIC best model).


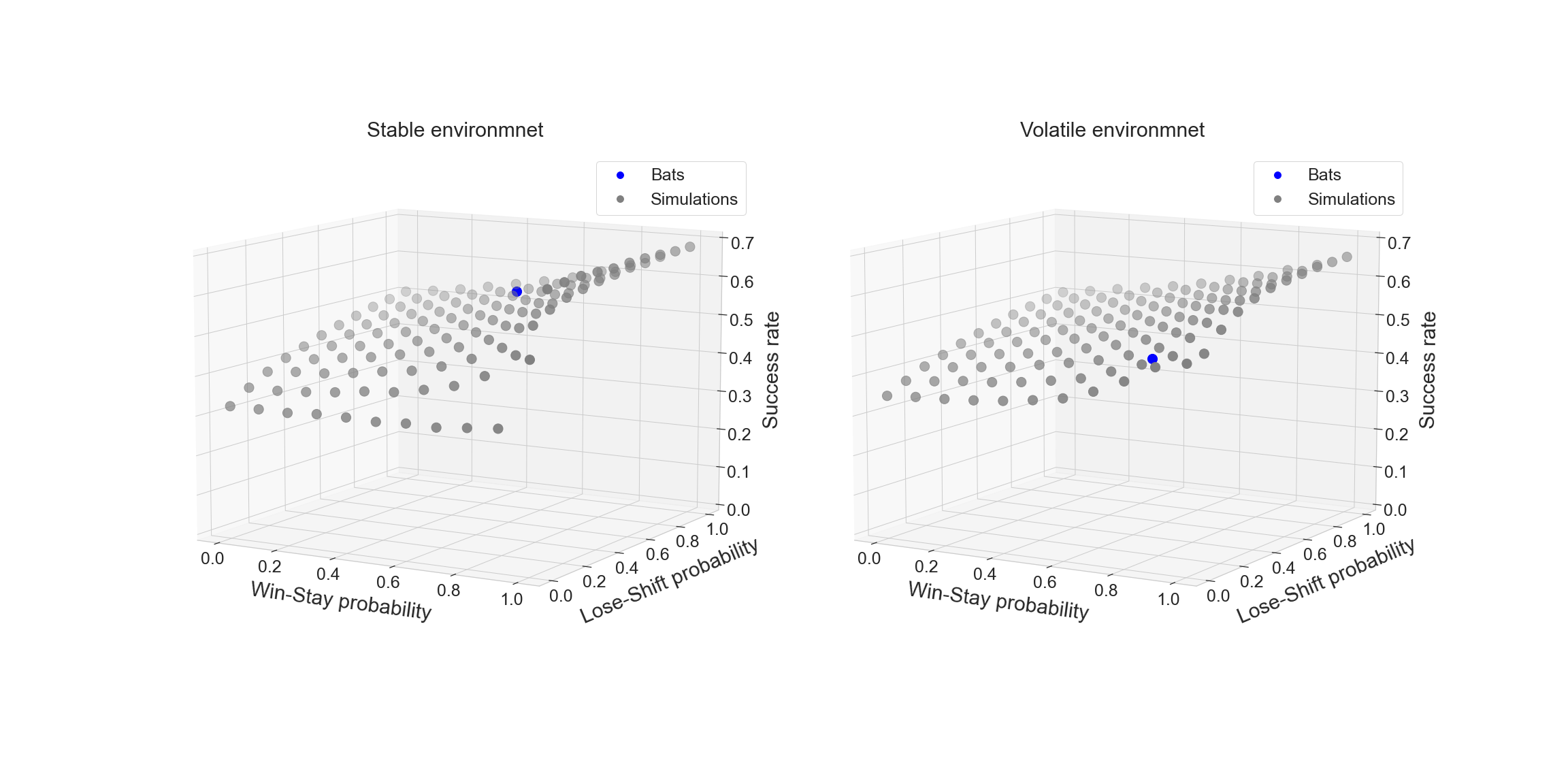


**Figure S1: WSLS model simulations’ success rate.** Bats in the stable environment had a success rate of 0.61 while simulations yielded a success rate of 0.52. Bats in the volatile environment had a success rate of 0.44 while simulations yielded a success rate of 0.52.

WSLS model includes two parameters- Win-Stay probability and Lose-Shift probability (see Methods). We used model fitting to find which Win-Stay probability and Lose-Shift probability result in maximum log-likelihood given the bat’s real behavior. We ran simulations for both environments (see Methods).

Bats in the volatile environment reached success rate of 0.44 in comparison to success rate of 0.52 yielded by simulations with similar Win-Stay-Lose-Shift probabilities as the bats, as extract from WSLS model fit. The optimal WSLS strategy simulation in volatile environment reached success rate of 0.65. Bats in the stable environment reached success rate of 0.61 in comparison to 0.52 yielded by simulations. Stable environment optimal WSLS simulation reached success rate of 0.68.
